# PotholeBAF-Net: Boundary-Aware Fusion Network with Low-Rank Transformer Bridge for Zero-Shot Cross-Dataset Pothole Segmentation

**DOI:** 10.3390/s26154796

**Published:** 2026-07-28

**Authors:** Maha Mesfer Alghamdi, Yakoop Qasim

**Affiliations:** 1 Department of Computer Science and Engineering, College of Applied Studies, King Saud University, Riyadh, Saudi Arabia; 2 Department of Mechatronics and Robotics Engineering, Taiz University, Taiz, Yemen; yakoop.qasim@taiz.edu.ye

**Keywords:** pothole segmentation, semantic segmentation, boundary-aware fusion, rank-bounded attention, domain generalization, zero-shot evaluation, road damage detection

## Abstract

Cross-dataset pothole segmentation is difficult because illumination, camera characteristics, asphalt texture, and boundary quality vary between training and deployment environments. This study proposes PotholeBAF-Net, an EfficientNet-B3 encoder–decoder network comprising a Low-Rank Transformer Bridge, a Boundary-Aware Fusion (BAF) decoder, and a lightweight Boundary Refinement Head. The bridge uses standard multi-head attention whose pre-softmax per-head affinity matrix is rank-bounded by the query/key dimension; BAF then regulates each encoder skip using a learned soft boundary prior, channel–spatial attention, and spatial–channel gated interpolation with decoder semantics. The model was trained and validated on the Large Public Dataset and evaluated without target-domain training or calibration on the independently collected 188-image Collected Taiz Dataset. Across three independent seeds, PotholeBAF-Net obtained external-test precision of 0.8562±0.1040, recall of 0.4854±0.0716, F1/Dice of 0.6196±0.0395, IoU of 0.4164±0.0365, and MCC of 0.5802±0.0198. Removing the boundary prior, Transformer bridge, or refinement head reduced mean IoU by 0.0763, 0.0685, and 0.0675, respectively. The network contains 12.463 M parameters and requires 4.779 GMACs. These findings support controlled skip transfer as a useful design for target-free pothole segmentation, while the remaining seed variability and recall-dominated failures show that shadows, weak boundaries, and visually ambiguous pavement remain unresolved challenges.

## 1. Introduction

Potholes are a persistent road-safety and infrastructure-management problem. Beyond reducing ride quality and accelerating vehicle deterioration, they can contribute to sudden maneuvers, loss of control, and traffic disruption [1,2,3]. Conventional inspection is labor-intensive, time-consuming, and difficult to apply consistently across large road networks [1,2]. Vision-based assessment offers a scalable alternative, and semantic segmentation is particularly useful because it estimates the complete damaged region rather than only a bounding box, thereby supporting localization, shape analysis, and area-based maintenance decisions [4,5,6,7,8].

The principal deployment challenge is reliable operation after the imaging domain changes. Road material, surface texture, illumination, shadows, moisture, camera characteristics, viewing geometry, pothole scale, and annotation style can differ substantially across datasets. Dark stains, repaired asphalt, cracks, and cast shadows may also resemble pothole regions. Unsupervised domain-adaptation methods can mitigate such shifts when target-domain samples are available [9,10,11,12,13]; however, target images may be unavailable before deployment or unsuitable for model calibration. The target-free setting considered here is, therefore, closer to single-source domain generalization: no target image is used for training, validation, threshold selection, fine-tuning, or post-processing calibration [14,15,16,17].

A related architectural limitation occurs in encoder–decoder networks. Skip connections restore spatial detail lost during downsampling, but they can also transmit source-specific texture responses directly into the decoder. Attention, Transformer context modeling, and boundary-aware supervision improve feature selectivity or contour quality [18,19,20,21,22,23,24,25,26]; nevertheless, they do not necessarily regulate, at every decoder stage, the relative contribution of boundary-relevant encoder detail and higher-level decoder semantics. Under domain shift, unrestricted low-level features may strengthen responses to shadows, stains, and pavement patterns even when the global context does not support a pothole prediction.

To address this gap, PotholeBAF-Net combines an EfficientNet-B3 encoder, a Low-Rank Transformer Bridge, a four-stage BAF decoder, and a lightweight Boundary Refinement Head. The central novelty is stage-wise regulation of skip-feature transfer rather than the isolated use of attention or boundary cues. At each decoder stage, a learned soft boundary prior and lightweight channel–spatial attention precondition the encoder feature. A spatial–channel gate then performs pointwise interpolation between the refined skip and decoder representations, allowing semantic context to suppress unreliable detail while retaining boundary evidence. The refinement head subsequently improves local mask consistency using residual depthwise-separable operations.

The main contributions are as follows:

- A BAF decoder that explicitly regulates skip transfer through boundary-conditioned enhancement, channel–spatial attention, and spatial–channel gated interpolation with decoder semantics. Unlike fixed concatenation or addition, the mechanism learns where and in which channels encoder detail should be retained or suppressed.
- A compact Transformer bridge whose pre-softmax per-head attention affinity is rank-bounded by the query/key dimension. Applied to 144 bottleneck tokens, it supplies global road-scene context without full-resolution attention; the precise scope of the rank claim is established in Section 3.5.2.
- A lightweight residual depthwise-separable refinement design integrated with the regulated decoder output to improve spatial consistency while maintaining a practical parameter and computation budget.

The remainder of the paper is organized as follows. Section 2 critically reviews pothole segmentation, boundary-aware learning, and cross-domain robustness. Section 3 presents the datasets, architecture, theoretical formulation, training protocol, and evaluation criteria. Section 4 reports the quantitative, statistical, computational, ablation, and failure-case analyses. Section 5 concludes the paper and identifies limitations and future directions.

## 2. Related Work

Related work is organized around the limitations most relevant to this study: dense pothole localization, boundary preservation and skip transfer, and generalization without target-domain access. This structure distinguishes methods that improve representation quality from methods that directly address the deployment protocol considered here.

### 2.1. Traditional, Sensor-Based, and Three-Dimensional Pothole Detection

Classical RGB methods combine thresholding, texture analysis, clustering, and morphology, but their fixed appearance assumptions are unreliable under shadows, wet asphalt, repairs, and heterogeneous aggregate [27,28,29,30]. Accelerometer systems are inexpensive but observe only the wheel path and depend on vehicle dynamics [31]. Stereo, RGB-D, LiDAR, structure-from-motion, and point-cloud methods can estimate depth or severity, yet require additional sensors, acquisition, and calibration [32,33,34,35,36,37]. These trade-offs motivate low-cost RGB segmentation while also explaining why monocular appearance alone cannot fully resolve geometrically ambiguous defects.

### 2.2. Deep Learning-Based Detection and Semantic Segmentation

Object detectors localize defects efficiently but do not provide the pixel-level area and contour needed for quantitative repair planning [38,39,40]. Dense architectures such as U-Net, DeepLabV3+, PSPNet, FPN, U-Net++, LinkNet, and SegFormer established complementary approaches to skip recovery, multi-scale context, and Transformer encoding [7,8,21,41,42,43,44,45]. Pothole-specific extensions have introduced Canny guidance, graph attention, or CNN–Transformer fusion [18,19,20,46]. These studies demonstrate the value of edges and context, but most report within-dataset performance. They, therefore, do not establish whether high-resolution skips preserve transferable defect geometry or primarily reproduce dataset-specific road texture.

### 2.3. Attention, Transformer, and Boundary-Aware Segmentation

Channel, spatial, squeeze-and-excitation, CBAM, and graph attention emphasize informative activations, but they do not by themselves specify how a decoder should balance semantic and skip streams [20,22,23,47]. Transformer models provide long-range context, although full-resolution attention is expensive and global reasoning alone does not delineate weak contours [21,48,49]. Boundary-aware segmentation has followed several distinct strategies. Boundary-Aware Feature Propagation restricts feature propagation using learned region boundaries [25]; InverseForm introduces a structured boundary-aware loss [24]; and Contextrast uses boundary-aware negative sampling within multi-scale contrastive learning [26]. Infrastructure-defect studies additionally use fixed Canny cues, learned boundary branches, gradient objectives, or final refinement modules [18,50,51].

These approaches confirm that contour information is useful, but their control points differ from the proposed decoder. Loss-based methods shape training without explicitly regulating the skip pathway; edge preprocessing depends on a fixed detector; and final refinement acts after encoder information has already entered the decoder. PotholeBAF-Net instead uses a learned soft prior to modulate each skip before fusion and then gates the refined skip against decoder semantics at every stage. The claimed advancement is, therefore, controlled skip transfer, not boundary awareness in isolation.

### 2.4. Domain Adaptation and Cross-Dataset Generalization

Unsupervised domain adaptation (UDA) assumes access to unlabeled target images and commonly uses image translation, stylization, self-training, or adversarial alignment [11,12]. In infrastructure inspection, Apedo et al. combine cross-domain stylization with dual adversarial feature learning for crack segmentation [13], while sim-to-real road-quality analysis uses segmentation-aware translation and alignment [10]. These studies are directly relevant to appearance shift, but their target access differs fundamentally from the present protocol.

Domain-generalized semantic segmentation (DGSS) instead trains without target-domain data [14]. Semantic-Aware Domain Generalized Segmentation separates domain-specific texture from semantic content [16]; WildNet diversifies source content and style using external wild imagery [17]; and recent foundation-model frameworks combine robust visual encoders, generative diversification, and mask refinement [15]. These methods show that source-only robustness can be improved through training-time diversification or external priors. The present study does not claim a general DG training algorithm and uses neither target images nor auxiliary foundation-model data. It asks a narrower architectural question: whether boundary-conditioned regulation of source-trained skip features improves target-free pothole segmentation.

### 2.5. Research Gap and Motivation

Three gaps follow from this analysis. First, pothole contours require learned boundary reasoning rather than fixed edge preprocessing alone. Second, high-resolution skips should be selectively transferred because they contain both useful contour evidence and source-specific texture. Third, evaluation should exclude target images from learning and calibration when the intended claim is target-free generalization. PotholeBAF-Net addresses these gaps through rank-bounded bottleneck context, boundary-modulated skips, adaptive fusion, and a strict external protocol.

## 3. Materials and Methods

This section presents the methodological framework used for pothole segmentation under a strict zero-shot cross-dataset setting. All models are trained and validated on a public source dataset and externally tested on an independently collected target dataset. No target-domain images are used for training, validation, checkpoint selection, threshold calibration, fine-tuning, or post-processing calibration. The framework includes problem formulation, dataset preparation, annotation processing, preprocessing and augmentation, the proposed PotholeBAF-Net architecture, baseline models, training strategy, and evaluation metrics.

### 3.1. Problem Formulation

Pothole segmentation is formulated as a binary semantic segmentation problem. Given an RGB road image, $X\in R^{3\times H\times W}$, the objective is to predict a pixel-wise mask, $Y\in {\left\{0,1\right\}}^{1\times H\times W}$, where foreground pixels correspond to pothole regions and background pixels correspond to the remaining road surface. The segmentation model $f_{\theta }\left(\cdot \right)$ produces a single-channel logit map, which is converted into a probability map using the sigmoid function:

(1) $$\hat{Y}=\sigma \left(f_{\theta }\left(X\right)\right),\;\hat{Y}\in {\left[0,1\right]}^{1\times H\times W}.$$

The final binary mask is obtained by applying a validation-selected threshold $\tau^{*}$:

(2) $$\hat{M}\left(p\right)=1\left[\hat{Y}\left(p\right)>\tau^{*}\right],$$

where $\hat{M}$ is the predicted mask, and 1[·] denotes the indicator function.

Let $D_{L}={\left\{\left(X_{i}^{L},Y_{i}^{L}\right)\right\}}_{i=1}^{N_{L}}$ denote the Large Public Dataset (LPD), which is used for training and validation, and let $D_{C}={\left\{\left(X_{j}^{C},Y_{j}^{C}\right)\right\}}_{j=1}^{N_{C}}$ denote the Collected Taiz Dataset (CTD), which is used only for external testing. The model parameters are learned by minimizing the segmentation loss over the LPD training split:

(3) $$\theta^{*}=\arg\min_{\theta }\frac{1}{N_{L}^{\mathrm{tr}}}\sum_{i=1}^{N_{L}^{\mathrm{tr}}}L\left(f_{\theta }\left(X_{i}^{L}\right),Y_{i}^{L}\right).$$

After training, the optimized model $f_{\theta^{*}}$ is evaluated on the CTD without using any CTD image during training, validation, threshold selection, fine-tuning, or post-processing. This protocol ensures that the external test results reflect cross-dataset generalization rather than target-domain adaptation.

### 3.2. Datasets and Cross-Dataset Evaluation Protocol

**Figure 1:**
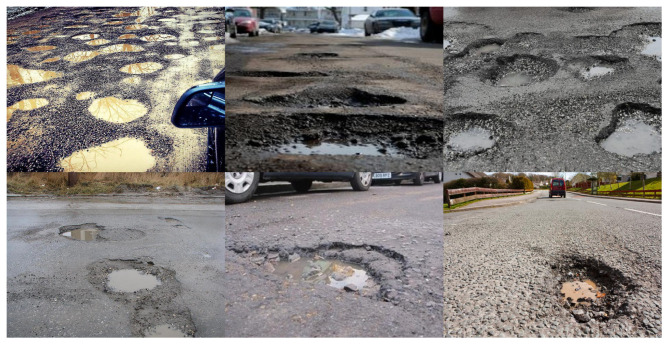
Representative samples from the Large Public Dataset (LPD).

Two datasets are used to evaluate pothole segmentation under source-to-target domain shift. The first dataset is the Pothole Segmentation YOLOv8 dataset obtained from Roboflow Universe [52]. In this study, it is referred to as the Large Public Dataset (LPD) and is used as the source domain for training and validation. The dataset contains RGB pothole images with a spatial resolution of 640×640 pixels. Its annotations are provided in YOLOv8 segmentation format as normalized polygon coordinates stored in TXT files. During data loading, these polygon annotations are rasterized into binary masks, where pothole regions are assigned to the foreground class, and all remaining pixels are assigned to the background class. The LPD is divided into 720 training images and 60 validation images. The public dataset record does not provide standardized camera-model, camera-to-road distance, or acquisition-height metadata; this missing information limits a controlled attribution of source-to-target differences. Representative LPD samples are shown in Figure 1.

**Figure 2:**
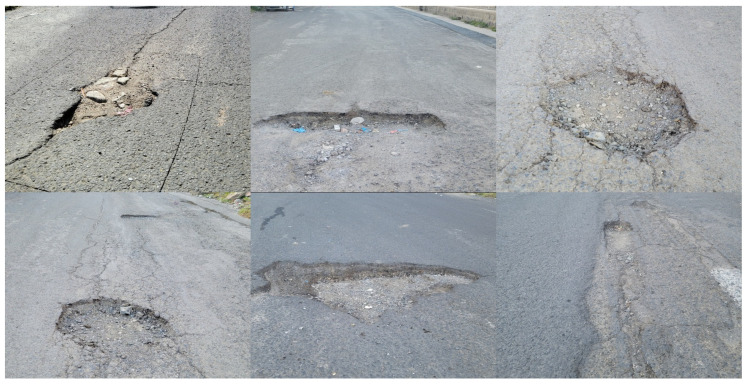
Representative samples from the Collected Taiz Dataset (CTD), which is used as an external target-domain test set.

The second dataset is the Collected Taiz Dataset (CTD), acquired specifically for this study on urban streets in Taiz, Yemen, and used only as the external target-domain test set. It contains 188 daytime RGB images captured with the 108 MP mode of a Samsung Galaxy S21 Ultra (Samsung, Suwon, Republic of Korea). The camera-to-pothole distance was not fixed or measured; the photographer varied the viewpoint and distance so that the complete pothole remained inside the frame. Consequently, CTD includes variation in apparent defect scale, perspective, illumination, asphalt texture, and boundary visibility. One annotator delineated each pothole as a polygon using LabelMe, after which an author visually reviewed every polygon mask against its source image and corrected evident omissions or boundary errors. The reviewed polygons were exported to normalized coordinates and rasterized into binary masks during evaluation. Representative samples are shown in Figure 2.

A strict target-free cross-dataset protocol is adopted. All models are trained and validated only on the LPD, while CTD is used exclusively for external testing. No CTD image is used for training, validation, checkpoint selection, hyperparameter tuning, threshold calibration, fine-tuning, or post-processing calibration. Although CTD was stored in two folders for file management, all 188 images are merged during inference and treated as one external set. This protocol is closer to single-source domain generalization than to unsupervised domain adaptation because target images are unavailable during learning.

### 3.3. Annotation Processing and Binary Mask Generation

Both datasets use polygon-based pothole annotations. For consistency, all annotations are converted into binary segmentation masks during data loading. For an image with original width, $W_{o}$, and height, $H_{o}$, each normalized polygon vertex, $v_{k}=\left(x_{k},y_{k}\right)$, is converted into pixel coordinates as

(4) $${\tilde{x}}_{k}=x_{k}W_{o},\;{\tilde{y}}_{k}=y_{k}H_{o}.$$

The resulting polygon is rasterized to form the binary mask:

(5) $$Y\left(p\right)=\left\{\begin{array}{cc} 1, & p\in P,\\ 0, & p\notin P, \end{array}\right.$$

where *P* denotes the filled pothole polygon region. If an image contains multiple pothole polygons, all regions are filled into the same mask. This binary representation is used consistently for training, validation, and external testing.

### 3.4. Preprocessing and Data Augmentation

All images and masks are resized to 384×384 pixels. RGB images are normalized using ImageNet statistics:

(6) $$X_{\mathrm{norm}}^{\left(c\right)}=\frac{X^{\left(c\right)}-\mu_{c}}{\sigma_{c}},$$

where μ=(0.485,0.456,0.406) and σ=(0.229,0.224,0.225) denote the ImageNet channel-wise mean and standard deviation values.

Data augmentation is applied only to LPD training images. Horizontal flip, vertical flip, and random 90° rotation are applied with probabilities 0.5, 0.2, and 0.3, respectively. Shift–scale–rotate augmentation (±8% translation, ±15% scaling, and ±20° rotation) is applied with probability 0.5. One of brightness–contrast adjustment (±0.2), CLAHE, or gamma correction (80–120) is selected with probability 0.6. One blur operation (Gaussian, motion, or median) and one color operation (hue–saturation–value shift, RGB shift, or grayscale conversion) are independently selected with probability 0.25; Gaussian noise is applied with probability of 0.20. These transformations expose the network to variations related to viewpoint, illumination, color response, shadow intensity, and road texture. No augmentation is applied during validation or CTD testing.

### 3.5. Proposed PotholeBAF-Net

The proposed PotholeBAF-Net is a boundary-aware encoder–decoder network designed for pothole segmentation under cross-dataset domain shift. The model is motivated by two practical challenges. First, pothole boundaries are often irregular, fragmented, weakly contrasted, and visually similar to surrounding asphalt. Second, road images contain confusing background patterns such as shadows, stains, cracks, repaired patches, loose gravel, and a heterogeneous texture. In conventional encoder–decoder networks, these low-level patterns may be transferred directly through skip connections, increasing false positives under source-to-target domain shift.

**Figure 3:**
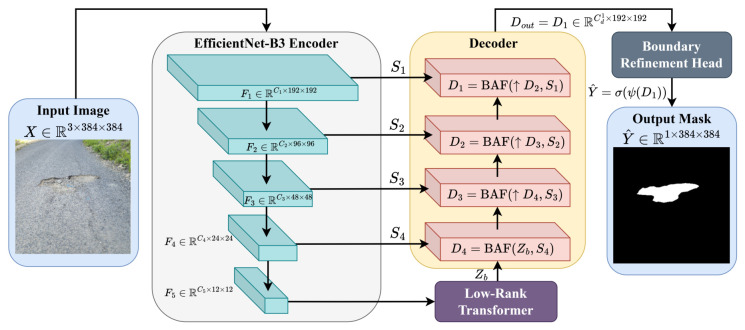
Architecture of the proposed PotholeBAF-Net segmentation model. The diagram shows the EfficientNet-B3 encoder, the Low-Rank Transformer Bridge, the Boundary-Aware Fusion decoder, and the Boundary Refinement Head.

To address this limitation, PotholeBAF-Net regulates encoder–decoder skip transfer instead of directly concatenating encoder and decoder features. The architecture consists of four components: an ImageNet-pretrained EfficientNet-B3 encoder, a Low-Rank Transformer Bridge, a Boundary-Aware Fusion (BAF) decoder, and a lightweight Boundary Refinement Head. The Transformer embedding dimension is 256. The four decoder stages use 192, 128, 80, and 48 output channels, with alternating dilation factors of 1 and 2 in their residual refinement blocks. The final head reduces the 48-channel decoder output to 24 channels before the one-channel logit projection. The complete model is expressed as

(7) $$\hat{Y}=\sigma \left(\psi \left(D_{\mathrm{BAF}}\left(T\left(E(X)\right)\right)\right)\right),$$

where E(·) is the EfficientNet-B3 encoder, T(·) is the Low-Rank Transformer Bridge, $D_{\mathrm{BAF}}\left(\cdot \right)$ is the proposed Boundary-Aware Fusion decoder, ψ(·) is the Boundary Refinement Head, and σ(·) is the sigmoid activation function. The overall architecture is shown in Figure 3.

#### 3.5.1. EfficientNet-B3 Encoder

The input image is first processed by an ImageNet-pretrained EfficientNet-B3 encoder to extract hierarchical multi-scale representations:

(8) $$\left\{F_{1},F_{2},F_{3},F_{4},F_{5}\right\}=E_{\theta_{e}}\left(X\right),$$

where $F_{1}$ to $F_{4}$ are used as skip features, and $F_{5}$ is passed to the Transformer bridge. For an input size of 384×384, the encoder produces feature maps at progressively reduced spatial resolutions, with the deepest feature map having an approximate resolution of 12×12. The skip features are denoted as

(9) $$S_{i}=F_{i},\;i=1,2,3,4.$$

This encoder design preserves local spatial detail in shallow layers while providing high-level semantic information in deeper layers.

#### 3.5.2. Low-Rank Transformer Bridge

Although CNN encoders are effective for local feature extraction, their local operators do not directly model long-range relationships. The bridge, therefore, applies two pre-norm Transformer encoder layers only to the 12×12 bottleneck. A 1×1 convolution projects $F_{5}$ to $C_{b}=256$ channels, producing N=144 tokens:

(10) $$T=\mathrm{Flatten}\left(\phi (F_{5})\right),\;T\in R^{N\times C_{b}},\;N=H_{5}W_{5},$$

where ϕ(·) denotes channel projection. The tokens are processed by a Transformer encoder and reshaped back into a spatial feature map:

(11) $$Z_{b}=R_{\mathrm{loc}}\left(\mathrm{Reshape}\left(\mathrm{TE}(T)\right)\right),$$

where TE(·) denotes the Transformer encoder, and $R_{\mathrm{loc}}\left(\cdot \right)$ is a residual depthwise-separable refinement with dilation of 2. Each Transformer layer uses four heads, a head dimension of $d_{h}=64$, a feed-forward width of 512, GELU activation, and dropout of 0.05. For head *h*, the pre-softmax attention affinity is

(12) $$L_{h}=\frac{Q_{h}K_{h}^{⊤}}{\sqrt{d_{h}}},\;Q_{h},K_{h}\in R^{N\times d_{h}},\;\mathrm{rank}\left(L_{h}\right)\leq d_{h}=64<N=144.$$

**Figure 4:**
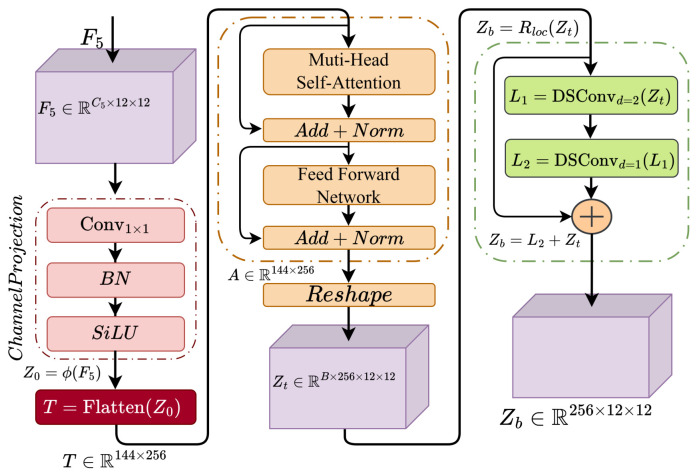
Low-rank Transformer bridge. Standard multi-head attention is applied to 144 bottleneck tokens with rank-bounded pre-softmax affinity logits, followed by local residual refinement.

Thus, “low-rank” refers specifically to the rank-bounded factorization of the pre-softmax affinity logits, while computational efficiency also follows from applying attention to 144 bottleneck tokens rather than $384^{2}$ image locations. The subsequent row-wise softmax is not claimed to preserve this rank bound, and the module is not a Linformer-style token-projection approximation. The bridge is used as a lightweight context component; the principal architectural novelty lies in the boundary-regulated skip fusion described next. Figure 4 summarizes the implementation.

#### 3.5.3. Boundary-Aware Fusion Decoder

The Boundary-Aware Fusion decoder is the main component of PotholeBAF-Net. Conventional decoders often concatenate skip and decoder features directly. Here, each skip is first refined using a learned boundary prior and channel–spatial attention and is then fused adaptively with the decoder feature through a learnable gate. This design aims to preserve boundary-relevant information while reducing the transfer of source-specific road texture.

The decoder contains four BAF stages. The Transformer bridge output $Z_{b}$ initializes the decoder, and the encoder skip features $S_{1},S_{2},S_{3},S_{4}$ are progressively fused as follows:

(13) $$\begin{array}{cc} D_{4} & =\mathrm{BAF}(Z_{b},S_{4}), \end{array}$$

(14) $$\begin{array}{cc} D_{3} & =\mathrm{BAF}(↑D_{4},S_{3}), \end{array}$$

(15) $$\begin{array}{cc} D_{2} & =\mathrm{BAF}(↑D_{3},S_{2}), \end{array}$$

(16) $$\begin{array}{cc} D_{1} & =\mathrm{BAF}(↑D_{2},S_{1}), \end{array}$$

where ↑ denotes bilinear upsampling by a factor of two. For the *i*-th BAF block, the decoder input and skip feature are denoted as $U_{i}$ and $S_{i}$, respectively.

The decoder feature is first projected to a common channel dimension:

(17) $$X_{i}=\phi_{x}\left(U_{i}\right),$$

where $\phi_{x}\left(\cdot \right)$ is implemented using a 1×1 convolution, followed by batch normalization and SiLU activation.

In parallel, the encoder skip feature is refined using two complementary operations. First, a soft boundary prior is estimated from the skip feature:

(18) $$E_{i}=\sigma \left(\phi_{e}\left(S_{i}\right)\right),$$

where $\phi_{e}\left(\cdot \right)$ consists of a 3×3 convolution, batch normalization, SiLU activation, and a 1×1 convolution. The map $E_{i}$ acts as a learned spatial guide that highlights regions likely to contain pothole boundaries.

Second, the skip feature is processed using lightweight channel–spatial attention:

(19) $${\bar{S}}_{i}=A_{\mathrm{cs}}\left(S_{i}\right),$$

where $A_{\mathrm{cs}}\left(\cdot \right)$ denotes the LiteCBAM attention module. The boundary prior and attention-refined skip feature are then combined as

(20) $${\hat{S}}_{i}={\bar{S}}_{i}⊙\left(1+E_{i}\right),$$

where ⊙ denotes element-wise multiplication. The term $\left(1+E_{i}\right)$ amplifies boundary-relevant responses while preserving the original skip information. The enhanced skip feature is then projected as

(21) $$S_{i}^{p}=\phi_{s}\left({\hat{S}}_{i}\right),$$

where $\phi_{s}\left(\cdot \right)$ is a 1×1 convolution followed by batch normalization and SiLU activation.

Instead of fixed concatenation, the proposed BAF block uses a learnable gate to balance decoder semantics and boundary-enhanced skip details:

(22) $$G_{i}=\sigma \left(\phi_{g}\left(\left[X_{i},S_{i}^{p}\right]\right)\right),$$

where $\left[X_{i},S_{i}^{p}\right]$ denotes channel-wise concatenation, and $\phi_{g}\left(\cdot \right)$ is implemented using a 1×1 convolution and batch normalization. The gated fusion output is computed as

(23) $$Q_{i}=G_{i}⊙X_{i}+\left(1-G_{i}\right)⊙S_{i}^{p}.$$

When $G_{i}$ is high, the block gives more weight to the decoder semantic feature; when $G_{i}$ is low, it gives more weight to the boundary-enhanced skip feature. The fused representation is finally refined using a residual depthwise-separable block and LiteCBAM:

(24) $$D_{i}=R_{i}\left(Q_{i}\right),$$

where $R_{i}\left(\cdot \right)$ denotes the local residual refinement module.

The complete BAF operation can be summarized as

(25) $$D_{i}=R_{i}\left(G_{i}⊙X_{i}+\left(1-G_{i}\right)⊙S_{i}^{p}\right),$$

with

(26) $$\begin{array}{cc} X_{i} & =\phi_{x}\left(U_{i}\right), \end{array}$$

(27) $$\begin{array}{cc} S_{i}^{p} & =\phi_{s}\left(A_{\mathrm{cs}}\left(S_{i}\right)⊙\left(1+\sigma (\phi_{e}\left(S_{i}\right))\right)\right), \end{array}$$

(28) $$\begin{array}{cc} G_{i} & =\sigma \left(\phi_{g}\left(\left[X_{i},S_{i}^{p}\right]\right)\right). \end{array}$$

Because the sigmoid gives $0<G_{i}\left(c,p\right)<1$ for channel *c* and location *p*, Equation (23) is a pointwise convex interpolation of the two projected streams. Before the nonlinear refinement $R_{i}$, each fused activation, therefore, lies between the corresponding decoder and skip activations:

(29) $$\min\left\{X_{i}\left(c,p\right),S_{i}^{p}\left(c,p\right)\right\}\leq Q_{i}\left(c,p\right)\leq \max\left\{X_{i}\left(c,p\right),S_{i}^{p}\left(c,p\right)\right\}.$$

This bounded mixture provides a clearer mechanism than unconstrained concatenation: the gate can reduce the direct contribution of a skip response without requiring the subsequent convolution to first disentangle two stacked streams. The gate is data-dependent and learned jointly with the segmentation loss; it is not asserted to guarantee domain invariance or shadow removal.

**Figure 5:**
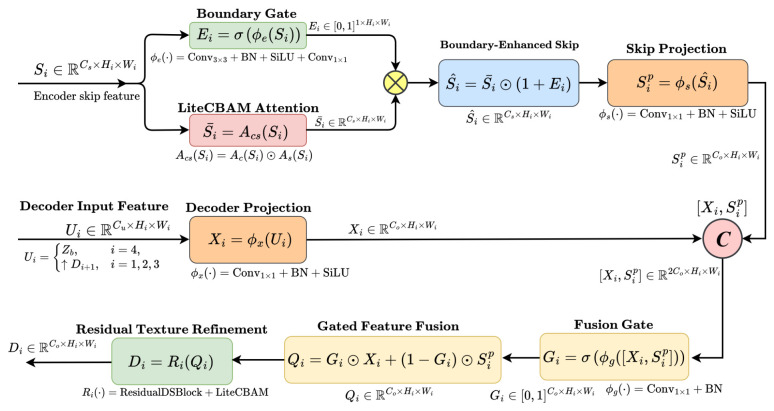
Detailed architecture of the proposed Boundary-Aware Fusion (BAF) block.

The formulation differs from direct concatenation because both streams are first projected to a common dimension, the skip is modulated by a learned boundary prior and channel–spatial attention, and the mixture is selected independently at each channel and spatial location. It can, therefore, attenuate source-specific texture when decoder context does not support it while retaining boundary evidence where the two streams agree. The detailed BAF block is shown in Figure 5.

#### 3.5.4. Boundary Refinement Head

The final decoder feature $D_{1}$ contains high-resolution information, but it may still include noisy responses from shadows, stains, cracks, repaired patches, or heterogeneous asphalt texture. Therefore, a lightweight Boundary Refinement Head is used to improve local mask consistency before producing the final pothole probability map. The refinement head applies a residual depthwise-separable block followed by an additional lightweight convolutional refinement:

(30) $$X_{r}=\mathrm{RDS}\left(D_{1}\right),\;X_{h}=\eta \left(X_{r}\right),$$

where RDS(·) denotes the residual depthwise-separable block and η(·) denotes a DSConvBNAct operation. Each depthwise-separable convolution is defined as

(31) $$\mathrm{DSConv}\left(X\right)=\delta \left(\mathrm{BN}\left({\mathrm{PW}}_{1\times 1}\left(\delta \left(\mathrm{BN}\left({\mathrm{DW}}_{3\times 3}\left(X\right)\right)\right)\right)\right)\right),$$

where ${\mathrm{DW}}_{3\times 3}$ denotes depthwise convolution, ${\mathrm{PW}}_{1\times 1}$ denotes pointwise convolution, BN denotes batch normalization, and δ(·) denotes the SiLU activation function.

The refined feature is projected into a single-channel logit map and upsampled to the input resolution:

(32) $${\hat{Y}}_{\mathrm{logit}}={\mathrm{Up}}_{2\times }\left(\phi_{o}\left(X_{h}\right)\right),\;\hat{Y}=\sigma \left({\hat{Y}}_{\mathrm{logit}}\right),$$

where $\phi_{o}\left(\cdot \right)$ is a 1×1 output projection and ${\mathrm{Up}}_{2\times }\left(\cdot \right)$ denotes bilinear upsampling. The final binary mask is obtained using the validation-selected threshold $\tau^{*}$:

(33) $$\hat{M}\left(p\right)=1\left[\hat{Y}\left(p\right)>\tau^{*}\right].$$

**Figure 6:**
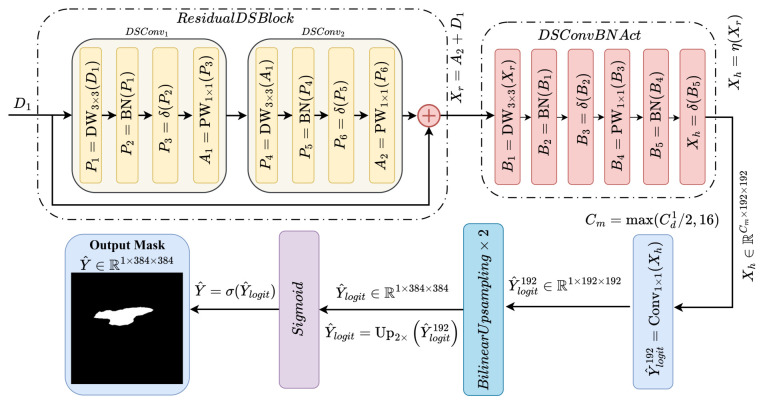
Detailed architecture of the proposed Boundary Refinement Head.

The Boundary Refinement Head improves the final prediction by applying residual local refinement, preserving decoder semantics, enhancing boundary-sensitive details, and maintaining low computational overhead. Its detailed architecture is shown in Figure 6.

### 3.6. Baseline Models

To evaluate PotholeBAF-Net, the baselines include U-Net, U-Net++, FPN, LinkNet, DeepLabV3+, PSPNet, MA-Net, and SegFormer. They represent direct and nested skip fusion, pyramid aggregation, atrous context, attention-based decoding, and Transformer encoding.

All models follow the same source-to-target split, preprocessing, optimizer, loss, augmentation, validation-based checkpoint and threshold selection, and metrics. The disclosed epoch-budget difference remains a comparison limitation; accordingly, the results evaluate the complete experimental configurations rather than isolating architecture under a perfectly matched training duration.

### 3.7. Loss Function

Pothole segmentation is affected by foreground–background imbalance because pothole regions usually occupy a much smaller area than the road background. Therefore, the training objective combines binary cross-entropy loss and focal Tversky loss:

(34) $$L_{\mathrm{total}}=\lambda_{\mathrm{BCE}}L_{\mathrm{BCE}}+\lambda_{\mathrm{FT}}L_{\mathrm{FT}},$$

where $L_{\mathrm{BCE}}$ provides stable pixel-wise supervision, and $L_{\mathrm{FT}}$ improves overlap-based foreground recovery.

The binary cross-entropy loss is defined as

(35) $$L_{\mathrm{BCE}}=-\frac{1}{\left|\Omega \right|}\sum_{p\in \Omega }\left[Y\left(p\right)\log\left(\hat{Y}\left(p\right)\right)+\left(1-Y\left(p\right)\right)\log\left(1-\hat{Y}\left(p\right)\right)\right],$$

where Ω denotes the set of image pixels, Y(p) is the ground-truth label, and $\hat{Y}\left(p\right)$ is the predicted pothole probability.

The Tversky index is computed as

(36) $$\mathrm{TI}=\frac{TP+s}{TP+\alpha FP+\beta FN+s},$$

where TP, FP, and FN denote true positives, false positives, and false negatives, respectively. The focal Tversky loss is then given by

(37) $$L_{\mathrm{FT}}={\left(1-\mathrm{TI}\right)}^{\gamma },$$

where γ is the focusing parameter, and *s* is used for numerical stability.

The loss-function hyperparameters are summarized in Table 1. A larger mixture weight is assigned to the focal Tversky term to emphasize region overlap under foreground–background imbalance. Within the Tversky denominator, α=0.7 places a larger penalty on false positives than β=0.3 places on false negatives.

### 3.8. Training Strategy and Threshold Selection

All models are trained using the AdamW optimizer with a cosine annealing learning-rate scheduler. Mixed-precision training is enabled when a CUDA-compatible GPU is available to reduce memory usage and improve computational efficiency. For each model, the checkpoint with the highest IoU on the LPD validation set is selected:

(38) $$\theta_{\mathrm{best}}=\arg\max_{\theta_{t}}{\mathrm{IoU}}_{\mathrm{LPD}}^{\mathrm{val}}\left(\theta_{t}\right),$$

where $\theta_{t}$ denotes the model parameters at epoch *t*.

After training, the best checkpoint is reloaded, and the decision threshold is selected only on the LPD validation set. The threshold is searched over τ∈[0.05,0.80] with a step size of Δτ=0.025, and the optimal threshold is selected as

(39) $$\tau^{*}=\arg\max_{\tau }\mathrm{IoU}\left(1\left[{\hat{Y}}^{\mathrm{val}}>\tau \right],Y^{\mathrm{val}}\right).$$

The selected threshold $\tau^{*}$ is then used for both LPD validation evaluation and CTD zero-shot testing. No threshold optimization or morphological post-processing is performed on the CTD, ensuring that the external test results reflect the direct segmentation ability of each model under the strict zero-shot protocol.

### 3.9. Experimental Setup and Reproducibility

The experimental framework is implemented in Python 3.10 using PyTorch 2.5.1. Image loading, polygon rasterization, and mask processing are performed using OpenCV, while data augmentation is implemented using Albumentations. CNN-based segmentation baselines and transformer-based components are implemented using PyTorch-compatible libraries and pretrained backbones.

All experiments are conducted on a Lenovo laptop equipped with an Intel Core i7-10750H CPU, 32 GB RAM, and an NVIDIA GeForce RTX 2060 GPU with 6 GB VRAM using CUDA 11.8. Automatic mixed precision is enabled. Independent runs use seeds 42, 123, and 2026; each seed is applied to Python, NumPy 2.2.6, and PyTorch, with deterministic cuDNN execution and benchmarking disabled. The proposed model, every ablation, and all baselines have three runs with different seeds.

All architectures, including the proposed model, its ablations, and all baseline models, were trained for a maximum of 40 epochs. They use the same input size, batch size, optimizer, learning-rate scheduler, loss function, data augmentation, validation protocol, threshold-selection procedure, and evaluation metrics. For every run, the checkpoint achieving the highest validation IoU on the LPD validation set was selected for evaluation. Thus, the training budget and checkpoint-selection criterion were consistent across all models. No target-domain images were used for training, validation, checkpoint selection, threshold selection, fine-tuning, or post-processing calibration. Table 2 summarizes the complete experimental configuration.

### 3.10. Performance Evaluation

Segmentation performance is evaluated using pixel-level metrics derived from true positives (TP), true negatives (TN), false positives (FP), and false negatives (FN). The primary metric is Intersection over Union (IoU), which measures the spatial overlap between the predicted pothole region and the ground-truth mask:

(40) $$\mathrm{IoU}=\frac{TP}{TP+FP+FN}.$$

Precision and recall are computed as

(41) $$\begin{array}{cc} \mathrm{Precision} & =\frac{TP}{TP+FP}, \end{array}$$

(42) $$\begin{array}{cc} \mathrm{Recall} & =\frac{TP}{TP+FN}. \end{array}$$

The F1-score is computed as

(43) $$F1=\frac{2\times \mathrm{Precision}\times \mathrm{Recall}}{\mathrm{Precision}+\mathrm{Recall}}.$$

The Dice coefficient is computed as

(44) $$\mathrm{Dice}=\frac{2TP}{2TP+FP+FN}.$$

The Matthews correlation coefficient (MCC) is also reported because it accounts for all four confusion-matrix components and is informative under class imbalance:

(45) $$\mathrm{MCC}=\frac{TP\cdot TN-FP\cdot FN}{\sqrt{\left(TP+FP)(TP+FN)(TN+FP)(TN+FN\right)}}.$$

For the principal validation, external-test, and ablation tables, confusion counts are pooled over all pixels in the evaluated dataset for each run; metrics are then reported as the mean ± sample standard deviation across valid seeds. F1 and Dice are algebraically identical under the binary definitions above, but both are retained to match common reporting conventions in the pothole literature.

To quantify source-to-target degradation, the validation-to-test generalization gap is computed for each metric M as

(46) $$\Delta_{M}=M_{\mathrm{val}}^{\mathrm{LPD}}-M_{\mathrm{test}}^{\mathrm{CTD}}.$$

where a smaller gap indicates stronger cross-dataset robustness.

For paired statistical analysis, a per-image Dice score is first computed for each CTD image and seed and then averaged across the valid seeds for that model. The full model and each single-component ablation are paired by the same 188 image names. The mean paired difference (full minus ablation) is accompanied by a 95% paired-bootstrap confidence interval using 5000 image-level resamples and a two-sided Wilcoxon signed-rank test. Holm adjustment is applied across the five component comparisons. This image-macro analysis weights every image equally and, therefore, need not equal the dataset-level pixel-pooled Dice in the principal tables.

Boundary-level overlays are used to examine contour structure qualitatively. Failure analysis uses the 20 images with the lowest full-model mean per-image IoU and reports their precision–recall pattern without assigning visual causes that cannot be verified from the images. This distinction is important because weak boundaries, shadows, heterogeneous asphalt, repairs, and small defects can produce similar metric signatures.

Finally, computational efficiency is assessed using the number of trainable parameters, floating-point operations (FLOPs), inference time, frames per second (FPS), and GPU memory usage. Parameters and FLOPs are computed using an input size of 384×384, while inference time and FPS are measured using batch size 1 and repeated runs to report mean and standard deviation.

## 4. Results and Discussion

This section presents the experimental results of PotholeBAF-Net and the baseline segmentation models under the adopted strict zero-shot cross-dataset protocol. All models were trained and validated on the Large Public Dataset (LPD), while external testing was performed on the independently collected Collected Taiz Dataset (CTD). No CTD image was used for training, validation, checkpoint selection, threshold selection, fine-tuning, or post-processing calibration. Therefore, the CTD results provide a realistic assessment of external-domain generalization rather than intra-dataset fitting.

### 4.1. Quantitative Performance on the LPD and CTD

Table 3 reports LPD validation performance over the available independent runs. PotholeBAF-Net achieves the highest mean IoU (0.7547±0.0098), F1/Dice (0.8650±0.0068), and MCC (0.8333±0.0088). FPN is the strongest baseline on mean IoU (0.7424±0.0225), although its estimate is based on two runs. The small source-domain margin motivates external evaluation rather than a claim based only on the in-distribution split.

Table 4 reports target-free external performance on CTD. The proposed model has the highest mean for all six metrics, but its variability is larger than on LPD, especially for precision, indicating sensitivity to initialization under domain shift.

To make the uncertainty of the external-test comparison explicit, approximate 95% confidence intervals were computed from the independent runs using the Student *t* distribution. Because the number of runs is small, these intervals are used as conservative uncertainty indicators rather than as definitive proof of superiority. For CTD IoU, PotholeBAF-Net obtained an approximate 95% confidence interval of 0.326–0.507, while FPN, the strongest baseline by mean IoU, obtained 0.329–0.479. The overlap between these intervals indicates that the small IoU margin over FPN should be interpreted descriptively. However, PotholeBAF-Net maintains the highest mean value across all six reported CTD metrics, and the component-level paired analysis in Section 4.5 further supports the contribution of the regulated decoding pathway, particularly the boundary-prior component. PotholeBAF-Net exceeds the FPN mean by 0.0123 IoU and 0.0441 F1/Dice, while its recall is higher by 0.0123. Nevertheless, the overlapping uncertainty intervals and the limited number of independent runs prevent a strong claim of statistically significant superiority over FPN. The comparison is, therefore, interpreted descriptively: the proposed model provides the best observed overall metric profile, but the remaining variability confirms the difficulty of segmentation under unseen-road conditions.

### 4.2. Validation-to-Test Generalization Gap

**Figure 7:**
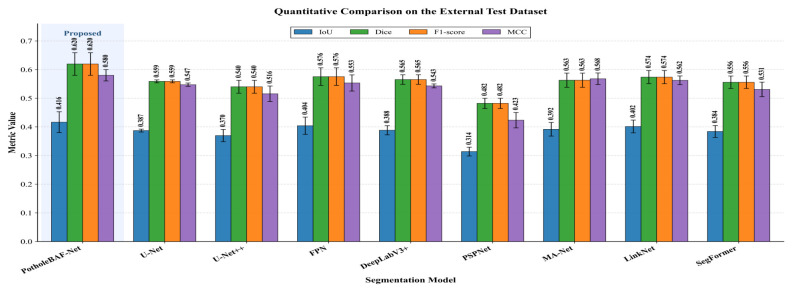
External-test IoU, Dice, F1, and MCC over available runs. Bars show means and error bars show sample standard deviations.

**Figure 8:**
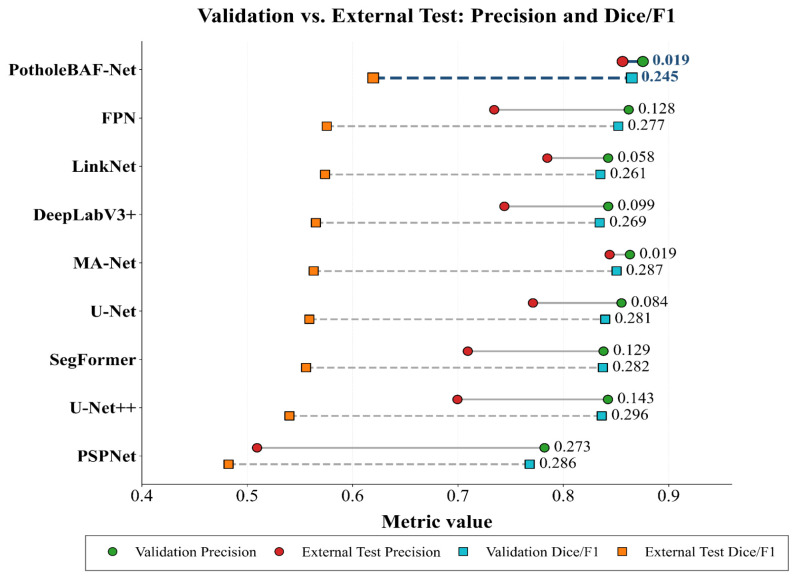
LPD validation versus CTD external-test precision and F1/Dice. Horizontal segments connect the domains and annotations give validation-minus-test gaps. Means use n=3 runs.

To examine domain shift without duplicating the four overlap metrics in Figure 7, Figure 8 compares precision and F1/Dice between LPD validation and CTD testing. Their gaps are

(47) $$\begin{array}{cc} \Delta_{\mathrm{Precision}} & ={\mathrm{Precision}}_{\mathrm{val}}^{\mathrm{LPD}}-{\mathrm{Precision}}_{\mathrm{test}}^{\mathrm{CTD}}, \end{array}$$

(48) $$\begin{array}{cc} \Delta_{F1} & =F1_{\mathrm{val}}^{\mathrm{LPD}}-F1_{\mathrm{test}}^{\mathrm{CTD}}. \end{array}$$

For PotholeBAF-Net, mean precision changes from 0.8755 to 0.8562 (gap 0.0193), whereas F1 decreases from 0.8650 to 0.6196 (gap 0.2454). The small precision change combined with the larger F1 change indicates that the external-domain difficulty primarily appears as missed pothole pixels rather than uncontrolled foreground expansion. FPN, U-Net++, and PSPNet show F1 gaps of approximately 0.277, 0.296, and 0.286, respectively. Thus, the proposed model retains the highest external F1, but the remaining recall loss is substantial and motivates the failure analysis below.

### 4.3. Qualitative and Boundary-Level Analysis

**Figure 9:**
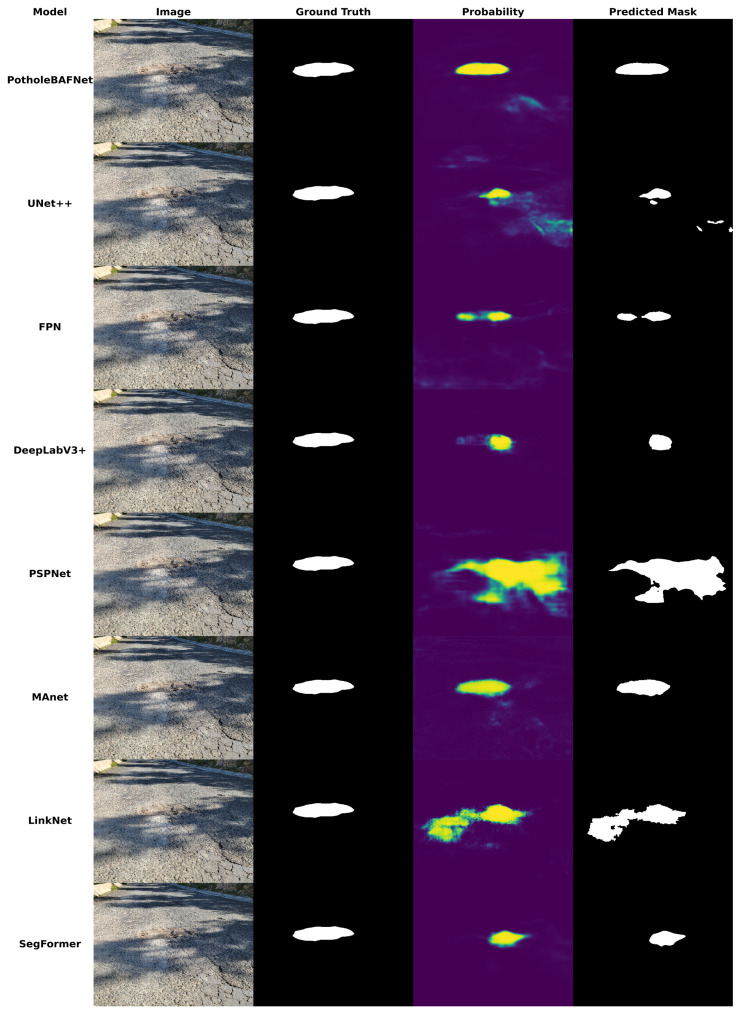
Qualitative CTD comparison under cast shadows. Columns show the input, ground truth, probability map, and thresholded prediction.

Figure 9 compares probability maps and binary predictions on a CTD image containing strong cast shadows. PotholeBAF-Net concentrates its highest response on the annotated defect, whereas several baselines activate on surrounding texture or fragment the mask. The BAF mechanism addresses shadows indirectly: photometric augmentation exposes the network to illumination changes, the boundary prior emphasizes spatial transitions, channel–spatial attention reweights skip responses, and the fusion gate can favor decoder context over a shadow-sensitive skip. None of these operations supplies an explicit shadow class, so the architecture attenuates rather than guarantees removal of shadow responses.

**Figure 10:**
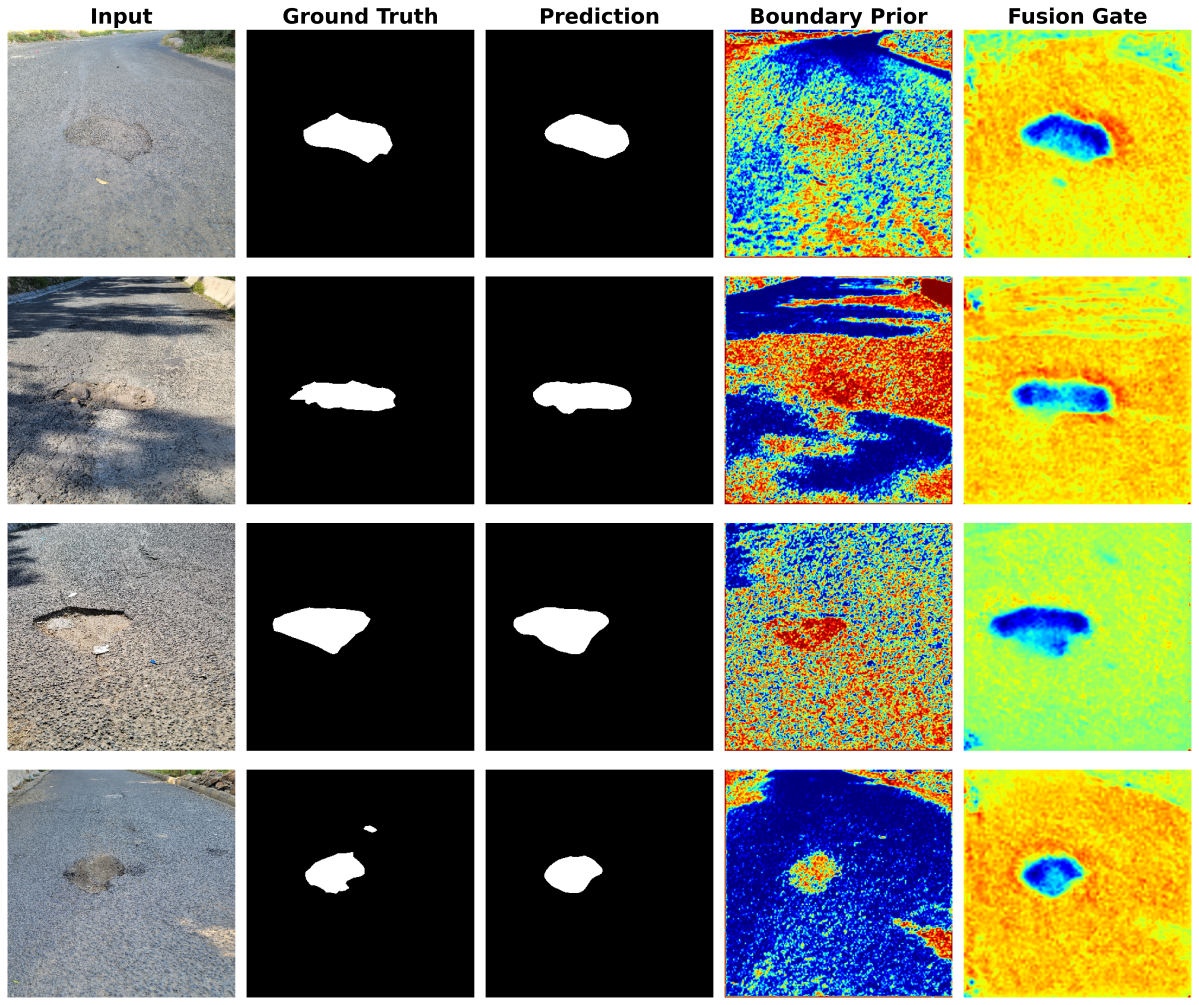
Visualization of learned Boundary-Aware Fusion behavior on representative CTD samples. Columns show the input image, ground-truth mask, predicted mask, learned boundary-prior map, and fusion-gate map. The boundary-prior maps highlight pothole contours and high-transition damaged regions, while the fusion-gate maps show spatially adaptive regulation of encoder skip transfer. Brighter gate values indicate stronger reliance on decoder semantic features, whereas darker values indicate stronger reliance on boundary-enhanced skip features.

To provide direct visual evidence of the behavior learned by the proposed BAF decoder, Figure 10 shows representative CTD samples together with the corresponding learned boundary-prior and fusion-gate maps. The boundary-prior maps exhibit stronger responses around pothole contours, damaged regions, and high-transition road-surface structures, indicating that this branch learns a spatial guide related to boundary-sensitive skip information rather than applying uniform enhancement across the image. The fusion-gate maps are also spatially non-uniform. In these maps, brighter values indicate stronger reliance on decoder semantic features, whereas darker values indicate stronger reliance on the boundary-enhanced encoder skip stream. The gate responses, therefore, show that encoder details are not transferred uniformly; instead, the decoder adaptively regulates the contribution of local skip information according to the surrounding semantic context.

These visualizations support the role of BAF as a selective skip-fusion mechanism. They show that the decoder does not simply concatenate high-resolution encoder features with decoder features; rather, it learns where boundary-sensitive local evidence should be preserved and where skip information should be moderated by decoder semantics. This behavior is consistent with the quantitative ablation results, where removing the boundary prior and adaptive fusion gate reduces external CTD performance. At the same time, these maps are interpreted as qualitative diagnostic evidence rather than causal proof; they indicate how the trained network distributes boundary emphasis and skip regulation, but they do not by themselves establish the physical cause of every success or failure case.

**Figure 11:**
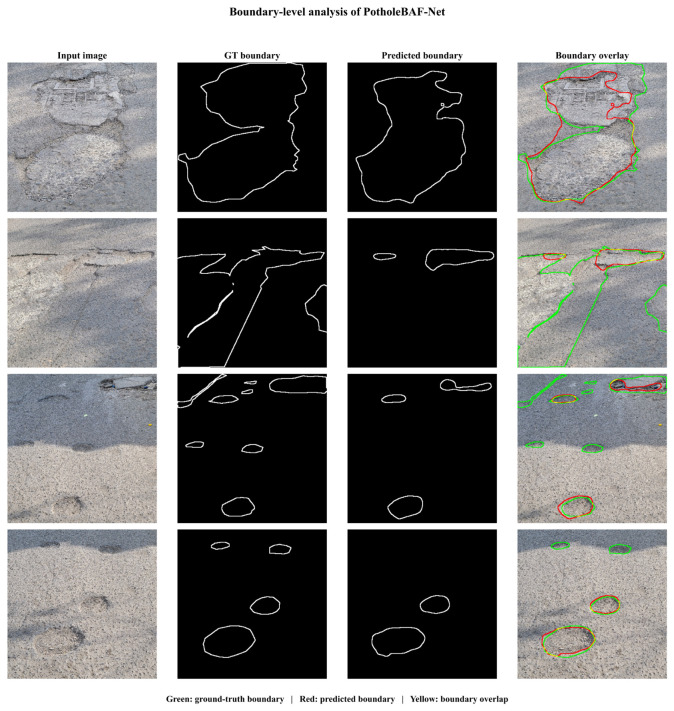
Boundary-level comparison between ground-truth masks and predicted masks on representative CTD samples. The comparison highlights the ability of PotholeBAF-Net to preserve the main pothole contour structure under external-domain testing.

Boundary-level analysis is further conducted to examine whether the predicted masks preserve pothole contour structure. This analysis is important because pothole boundaries are often weak, irregular, fragmented, and visually similar to surrounding asphalt. Figure 11 shows representative boundary-level comparisons between ground-truth masks and predicted masks on CTD samples.

The overlays show both successes and limitations. Coherent contours are recovered when the defect has a visible rim, whereas low-contrast or fragmented regions are smoothed or omitted. A metric-based audit of the 20 lowest-IoU CTD images further shows that 15 have precision greater than recall, four have recall greater than precision, and one is missed completely; false negatives, therefore, dominate most severe failures. For example, image A_train/20241024_064201.png has precision 0.9911 but recall 0.0707, whereas A_validation/20240722_091908.png has precision 0.0374 and recall 0.3095, representing a false-positive-dominated exception. The complete miss and very low-recall cases generally contain small, weak, or spatially diffuse annotations. These observations agree with the external precision–recall imbalance and prevent attributing all errors to one cause such as shadows.

The model has no semantic representation of “shadow” as an object. A possible extension is to use a vision–language model or world-model teacher to provide semantic nuisance concepts (shadow, stain, repair patch, and loose aggregate), followed by knowledge distillation into the lightweight segmenter. Such a system would require an additional dataset and controlled evaluation; it is, therefore, proposed as future work rather than claimed as part of the present method.

**Figure 12:**
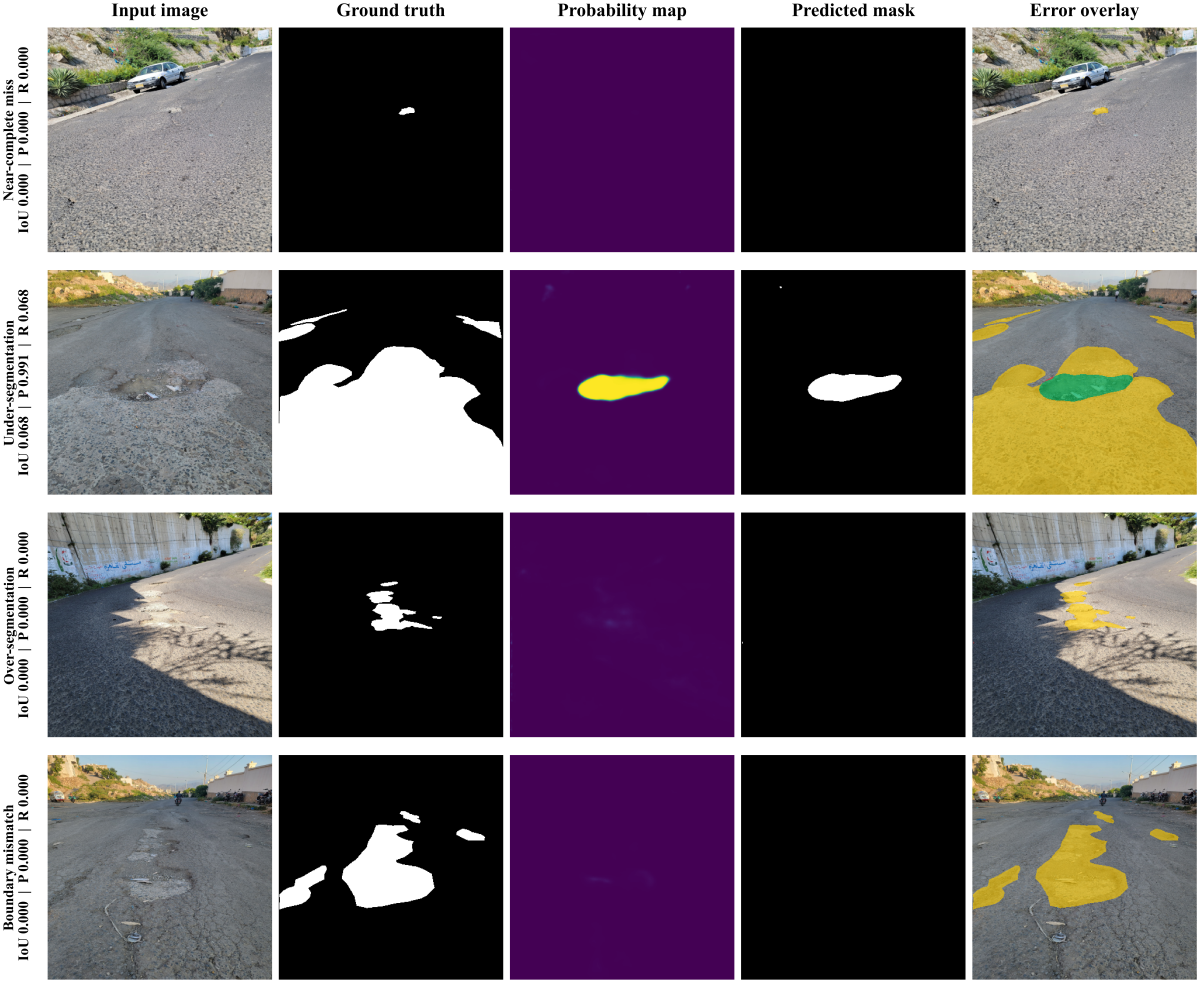
Representative severe failure cases on the external dataset. Columns show the input image, ground-truth mask, predicted probability map, thresholded mask, and error overlay. Rows illustrate a near-complete miss, under-segmentation, over-segmentation or false activation, and boundary mismatch. The labels report per-image IoU, precision (P), and recall (R).

Figure 12 complements the numerical audit with representative severe errors: a near-complete miss, strong under-segmentation, false activation on shadowed or textured pavement, and a boundary-mismatch case. These examples show that high precision on some images can coexist with substantial missed pothole area, whereas other scenes produce spatially misplaced responses.

### 4.4. Ablation and Component Interaction Analysis

Table 5 reports three-seed CTD results for single-component removals and interaction configurations. All five removals reduce dataset-level mean IoU relative to the complete network. The largest decreases occur without the boundary prior (0.0763), Transformer bridge (0.0685), and Boundary Refinement Head (0.0675). Removing channel–spatial attention or the adaptive fusion gate produces smaller decreases of 0.0280 and 0.0317. Because F1 and Dice are algebraically identical for binary masks under the reported definition, their columns match; both are retained for consistency with the main tables.

**Figure 13:**
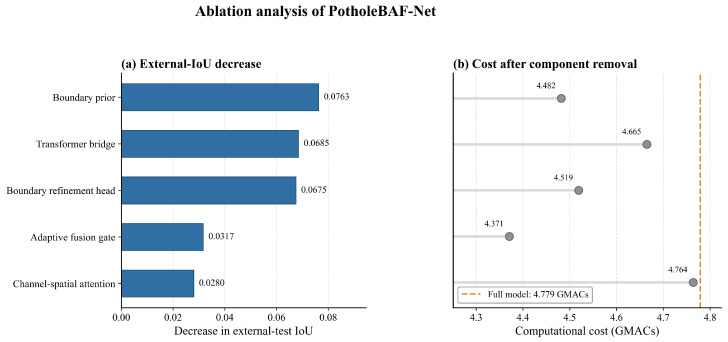
Component-removal analysis. (**a**) Decrease in mean external IoU relative to the full model. (**b**) GMACs of each ablated configuration; the dashed line marks the full-model cost.

The modules are not additive in isolation. A basic encoder–decoder obtains 0.3940±0.0319 IoU; adding only the Transformer gives 0.3824±0.0201, and adding only BAF gives 0.3693±0.0170, whereas their coordinated use with refinement reaches 0.4164±0.0365. This interaction indicates that global context, controlled skip transfer, and final local refinement function as a coupled decoding pathway rather than independent plug-ins. Figure 13 relates the performance decreases to cost. The full model requires 4.779 GMACs; the boundary prior, Transformer, and refinement-head removals require 4.482, 4.665, and 4.519 GMACs, respectively. The largest IoU gains, therefore, do not simply follow the largest computation increase.

### 4.5. Paired Statistical Analysis

Table 6 reports the paired image-macro Dice analysis defined in Section 3.10. The boundary prior has the clearest independent effect: its removal decreases mean per-image Dice by 0.0358, the bootstrap interval excludes zero, and the comparison remains significant after Holm adjustment. The unadjusted Transformer and refinement-head comparisons are significant, but their Holm-adjusted *p*-values are 0.0618 and 0.0661, respectively. Removing channel–spatial attention or the adaptive gate does not significantly reduce image-macro Dice in isolation.

The statistical and dataset-level ablation summaries answer different questions. Table 5 pools all CTD pixels within each seed and shows lower mean IoU for every removal. Table 6 weights each image equally and shows that the smaller channel-attention and gate effects are not independently robust after multiplicity correction. Together with the interaction configurations, this result supports a coupled-pathway interpretation and avoids attributing the full improvement to every module separately.

### 4.6. Computational Complexity and Inference Efficiency

Table 7 distinguishes multiply–accumulate operations (GMACs) from profiler FLOPs. PotholeBAF-Net contains 12.463 M parameters, requires 4.779 GMACs (9.557 profiler GFLOPs), and uses 140.6±5.8 MB peak GPU memory. It is less computation-intensive than U-Net, U-Net++, FPN, DeepLabV3+, and MA-Net, but more intensive than PSPNet and LinkNet; SegFormer has substantially fewer parameters.

**Figure 14:**
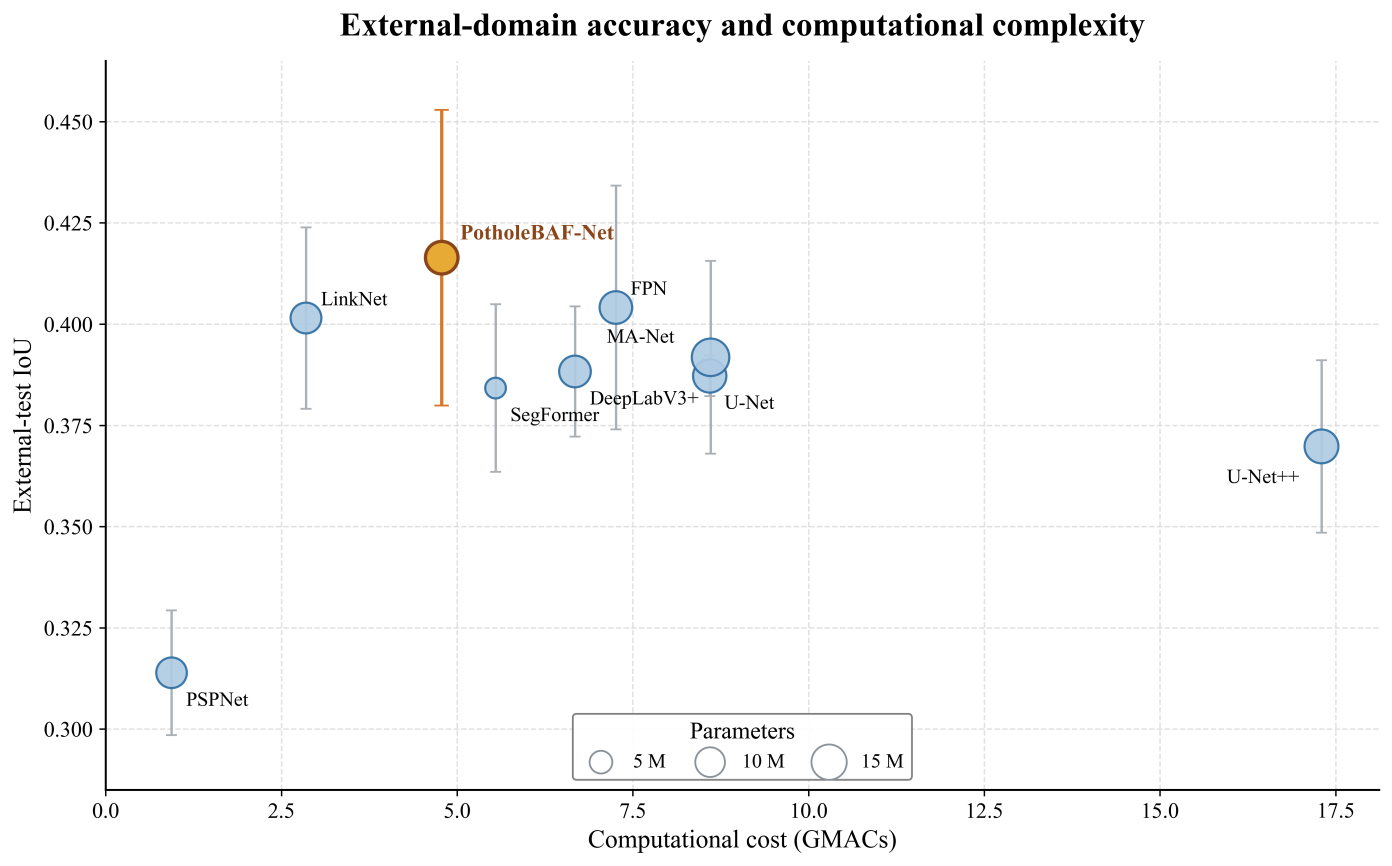
External-test IoU versus GMACs. Marker area represents parameter count and vertical error bars show sample standard deviation over available runs (n=3 except FPN, n=2).

The proposed model records 95.10±53.86 ms latency and 15.41±12.97 FPS. The large variation is caused by the constrained laptop-GPU measurement environment and precludes a strong real-time claim. PSPNet and SegFormer are faster but have a lower external IoU. Figure 14, therefore, presents the result as an accuracy–complexity trade-off, not as universal dominance in efficiency. Deployment latency should be re-profiled on the intended hardware with a standardized warm-up and timing protocol.

### 4.7. Overall Discussion

The available-run results support two main interpretations. First, the proposed network preserves strong source performance and has the highest observed external mean for all six reported metrics. The small IoU margin over FPN remains descriptive because FPN has only two valid runs and the uncertainty bands overlap. Second, the improvement is associated with the complete decoding pathway rather than one isolated plug-in: removing the boundary prior, Transformer, or refinement head causes the largest dataset-level IoU decreases, while adding only the Transformer or BAF to a basic decoder does not reproduce the full result. Global context appears most useful when paired with regulated high-resolution transfer and local refinement.

The improved external behavior of PotholeBAF-Net can be explained by controlled feature transfer under domain shift. In cross-dataset pothole segmentation, high-resolution encoder skips are necessary for recovering irregular boundaries, but they also carry low-level appearance cues that may be dataset-specific, including asphalt granularity, illumination patterns, stains, shadows, and repaired-road texture. Direct skip concatenation can, therefore, pass both useful contour evidence and unreliable source-domain texture into the decoder. The proposed BAF pathway reduces this risk by conditioning the skip stream with a learned boundary prior and then using a spatial–channel gate to interpolate between boundary-enhanced local detail and decoder semantic context. This mechanism allows the decoder to preserve fine structure where local evidence agrees with the predicted pothole region while moderating skip responses that are less consistent with the higher-level representation. The observed generalization improvement is, therefore, interpreted as the result of regulated skip transfer and complementary bottleneck context, rather than as a simple increase in model capacity.

The paired image-macro analysis refines this interpretation. Only the boundary-prior removal remains significant after Holm correction; the Transformer and refinement-head comparisons are suggestive but do not pass the adjusted 0.05 threshold, and the smaller attention and gate effects are not independently significant. Thus, the evidence supports the complete regulated pathway and a particularly important boundary-prior contribution but not a claim that every component produces an independently significant gain.

The shadow example clarifies the mechanism and its boundary. BAF can attenuate an encoder response when decoder context disagrees, but the model has no explicit nuisance label and still fails on some shadows, repairs, stains, and low-contrast defects. The 20-case audit shows that severe failures are predominantly recall-limited. Thus, the principal remaining problem is incomplete recovery of ambiguous pothole extent, not merely false-positive texture propagation.

The evidence is limited by the 188-image external dataset, only three proposed-model runs, two FPN runs, one polygon annotator followed by author review, an unmeasured acquisition distance, missing acquisition metadata for the public source dataset, and mixed retained baseline epoch histories. The wide uncertainty intervals mean that small margins should not be generalized beyond the studied datasets. Computational measurements also show high latency variance on the laptop GPU. Future studies should use multiple external cities and annotators, a uniform training budget, repeated hardware profiling, explicit shadow/repair labels, and temporal, depth, uncertainty, or vision–language teacher information.

## 5. Conclusions

This study shows that encoder detail should not be transferred uncritically when deployment appearance differs from the source domain. PotholeBAF-Net operationalizes this principle by conditioning each skip on a learned boundary prior and attention, then interpolating it with decoder semantics. Across three seeds, the model achieved 0.4164±0.0365 IoU and 0.6196±0.0395 F1/Dice on an independently collected dataset excluded from all training and calibration. Component removal identifies the boundary prior, bottleneck context, and final refinement as the largest dataset-level contributors, whereas the interaction and multiplicity-adjusted analyses show that the modules should be interpreted as a coupled pathway rather than a collection of independently significant plug-ins.

The result is promising but not definitive. CTD contains only 188 images from one city, uses one annotator with author review, and was acquired without measured camera distance; FPN has two valid runs, baseline checkpoint histories are mixed, and severe failures are predominantly recall-limited. Future work will, therefore, prioritize uniform-budget multi-site and multi-annotator evaluation, explicit shadow and repair-patch labels, and temporal or depth cues. Vision–language or world-model teachers may also provide nuisance concepts such as shadow, stain, and repaired asphalt, but their value must be tested through controlled distillation experiments rather than assumed.

## Figures and Tables

**Table 1:** Loss-function hyperparameters used for training.

Parameter	Value	Description
$\lambda_{\mathrm{BCE}}$	0.3	Weight of binary cross-entropy loss
$\lambda_{\mathrm{FT}}$	0.7	Weight of focal Tversky loss
α	0.7	Penalty assigned to false positives
β	0.3	Penalty assigned to false negatives
γ	0.75	Focusing parameter of focal Tversky loss
*s*	1.0	Constant for numerical stability

**Table 2:** Experimental configuration used for all models.

Parameter	Value
Input size	384×384
Batch size	2
Initial learning rate	$3\times 10^{-4}$
Weight decay	$1\times 10^{-4}$
Optimizer	AdamW
Scheduler	Cosine annealing
Threshold range	[0.05,0.80]
Threshold step	0.025
Post-processing	False
Epoch budget	40 epochs
Random seeds	42, 123, and 2026
Transformer layers/heads	2/4
Embedding/FFN dimensions	256/512
Transformer dropout	0.05

**Table 3:** LPD source-validation performance over three independent runs (mean ± sample standard deviation; n=3 for every model). Bold values indicate the highest (best) performance.

Model	Precision	Recall	F1	IoU	Dice	MCC
PotholeBAF-Net	0.8755±0.0183	0.8547±0.0098	0.8650±0.0068	0.7547±0.0098	0.8650±0.0068	0.8333±0.0088
U-Net	0.8552±0.0062	0.8249±0.0173	0.8398±0.0076	0.7238±0.0112	0.8398±0.0076	0.8065±0.0084
U-Net++	0.8423±0.0169	0.8308±0.0260	0.8365±0.0055	0.7185±0.0081	0.8365±0.0055	0.8017±0.0058
FPN	0.8620±0.0160	0.8424±0.0137	0.8521±0.0148	0.7424±0.0225	0.8521±0.0148	0.8210±0.0180
DeepLabV3+	0.8426±0.0174	0.8267±0.0257	0.8346±0.0055	0.7156±0.0082	0.8346±0.0055	0.7995±0.0056
PSPNet	0.7821±0.0195	0.7546±0.0117	0.7681±0.0137	0.6236±0.0181	0.7681±0.0137	0.7197±0.0170
MA-Net	0.8632±0.0023	0.8382±0.0123	0.8505±0.0053	0.7398±0.0079	0.8505±0.0053	0.8192±0.0058
LinkNet	0.8425±0.0106	0.8280±0.0162	0.8352±0.0031	0.7167±0.0046	0.8352±0.0031	0.8003±0.0029
SegFormer	0.8382±0.0189	0.8371±0.0085	0.8376±0.0052	0.7204±0.0076	0.8376±0.0052	0.8029±0.0070

**Table 4:** Target-free CTD external-test performance over three independent runs (mean ± sample standard deviation; n=3 for every model). Bold values indicate the highest (best) performance.

Model	Precision	Recall	F1	IoU	Dice	MCC
PotholeBAF-Net	0.8562±0.1040	0.4854±0.0716	0.6196±0.0395	0.4164±0.0365	0.6196±0.0395	0.5802±0.0198
U-Net	0.7711±0.0394	0.4384±0.0191	0.5590±0.0052	0.3872±0.0050	0.5590±0.0052	0.5473±0.0059
U-Net++	0.6995±0.0426	0.4398±0.0187	0.5401±0.0225	0.3698±0.0213	0.5401±0.0225	0.5157±0.0271
FPN	0.7344±0.0109	0.4731±0.0368	0.5755±0.0306	0.4041±0.0301	0.5755±0.0306	0.5533±0.0282
DeepLabV3+	0.7440±0.0927	0.4557±0.0606	0.5652±0.0167	0.3883±0.0161	0.5652±0.0167	0.5432±0.0069
PSPNet	0.5093±0.0763	0.4581±0.0459	0.4823±0.0179	0.3139±0.0154	0.4823±0.0179	0.4235±0.0267
MA-Net	0.8439±0.0026	0.4225±0.0271	0.5631±0.0247	0.3918±0.0238	0.5631±0.0247	0.5679±0.0202
LinkNet	0.7847±0.0303	0.4524±0.0368	0.5739±0.0229	0.4015±0.0224	0.5739±0.0229	0.5625±0.0153
SegFormer	0.7093±0.0521	0.4571±0.0287	0.5559±0.0217	0.3842±0.0207	0.5559±0.0217	0.5308±0.0254

**Table 5:** Single-component ablation and interaction results on CTD across three independent seeds (mean ± sample standard deviation; n=3 for every configuration).

Configuration	Precision	Recall	F1	IoU	Dice	MCC
Full PotholeBAF-Net	0.8562±0.1040	0.4854±0.0716	0.6196±0.0395	0.4164±0.0365	0.6196±0.0395	0.5802±0.0198
Without Transformer bridge	0.7179±0.0992	0.4077±0.0353	0.5201±0.0132	0.3479±0.0120	0.5201±0.0132	0.5011±0.0282
Without boundary prior	0.8228±0.0362	0.3663±0.0482	0.5069±0.0530	0.3401±0.0477	0.5069±0.0530	0.5186±0.0497
Without channel–spatial attention	0.7971±0.0261	0.4317±0.0241	0.5601±0.0143	0.3884±0.0137	0.5601±0.0143	0.5543±0.0060
Without adaptive fusion gate	0.7435±0.0805	0.4494±0.0655	0.5602±0.0344	0.3847±0.0333	0.5602±0.0344	0.5395±0.0229
Without refinement head	0.7740±0.0493	0.3886±0.0693	0.5174±0.0679	0.3489±0.0615	0.5174±0.0679	0.5140±0.0598
Basic encoder–decoder	0.7328±0.1564	0.4705±0.0419	0.5731±0.0329	0.3940±0.0319	0.5731±0.0329	0.5453±0.0571
Basic + Transformer	0.7762±0.0499	0.4303±0.0207	0.5537±0.0208	0.3824±0.0201	0.5537±0.0208	0.5442±0.0251
Basic + BAF	0.6622±0.0714	0.4602±0.0507	0.5430±0.0181	0.3693±0.0170	0.5430±0.0181	0.5081±0.0176

**Table 6:** Paired comparison of full PotholeBAF-Net with single-component ablations on 188 CTD images. Δ Dice is full minus ablation after averaging each image over the three seeds. Confidence intervals use 5000 paired bootstrap resamples; *p* is from a two-sided Wilcoxon signed-rank test.

Comparison	Δ Dice	Bootstrap 95% CI	Raw *p*	Holm-Adjusted *p*
Without Transformer bridge	0.0176	[0.0037, 0.0320]	0.0154	0.0618
Without boundary prior	0.0358	[0.0215, 0.0502]	<0.0001	<0.0001
Without channel–spatial attention	−0.0093	[−0.0238, 0.0048]	0.2330	0.3695
Without adaptive fusion gate	−0.0087	[−0.0217, 0.0038]	0.1847	0.3695
Without refinement head	0.0125	[0.0004, 0.0245]	0.0220	0.0661

**Table 7:** Computational complexity and batch-one inference measurements at 384×384 (mean ± sample standard deviation where applicable).

Model	Params (M)	GMACs	Profiler GFLOPs	Time (ms)	FPS	Memory (MB)
PotholeBAF-Net	12.463	4.779	9.557	95.10±53.86	15.41±12.97	140.6±5.8
U-Net	13.159	8.594	17.189	31.33±4.12	32.32±4.59	146.3±0.5
U-Net++	13.625	17.298	34.595	28.49±1.74	35.19±2.08	175.9±0.5
FPN	12.475	7.259	14.519	34.49±0.00	29.00±0.00	144.2±0.0
DeepLabV3+	11.680	6.677	13.354	267.51±1.71	3.74±0.02	140.4±0.5
PSPNet	10.753	0.937	1.873	9.40±0.86	107.03±10.37	136.6±0.5
MA-Net	17.171	8.606	17.213	32.98±1.06	30.34±0.99	159.5±5.8
LinkNet	10.927	2.850	5.701	37.65±0.00	26.56±0.00	119.1±0.0
SegFormer	3.812	5.547	11.095	16.95±0.00	58.98±0.00	84.5±0.0

## Data Availability

The data used in this study are available online. The Collected Taiz Dataset (CTD), which was independently collected for external zero-shot evaluation, is available at: https://drive.google.com/drive/folders/1-QcTuilB92j6yI0ehI6ut-6TgRmQSBfk?usp=drive_link (accessed on 18 July 2026), The Large Public Dataset (LPD) used for training and validation is publicly available from Roboflow Universe [52].
